# Supplementary material for: Ectopic overproduction of cell wall glucan through membrane perturbation by an antifungal peptide theonellamide A in fission yeast
Source: iScience. 2026 Oct 1;29(10):117645. doi: 10.1016/j.isci.2026.117645 (PMC13657213; doi:10.1016/j.isci.2026.117645)
Supplement: Supplementary file 1 [file mmc1.pdf]

iScience, Volume *29*

Supplemental information

Ectopic overproduction of cell wall glucan through membrane perturbation by an antifungal peptide theonellamide A in fission yeast

Kensuke Nakao, Vanessa S.D. Carvalho, Akifumi Suganaga, Masako Osumi, Masato Tokukura, Hideaki Kakeya, Akihisa Matsuyama, Yoko Yashiroda, Shigeki Matsunaga, Juan C.G. Cortés, Minoru Yoshida, Juan C. Ribas, and Shinichi Nishimura

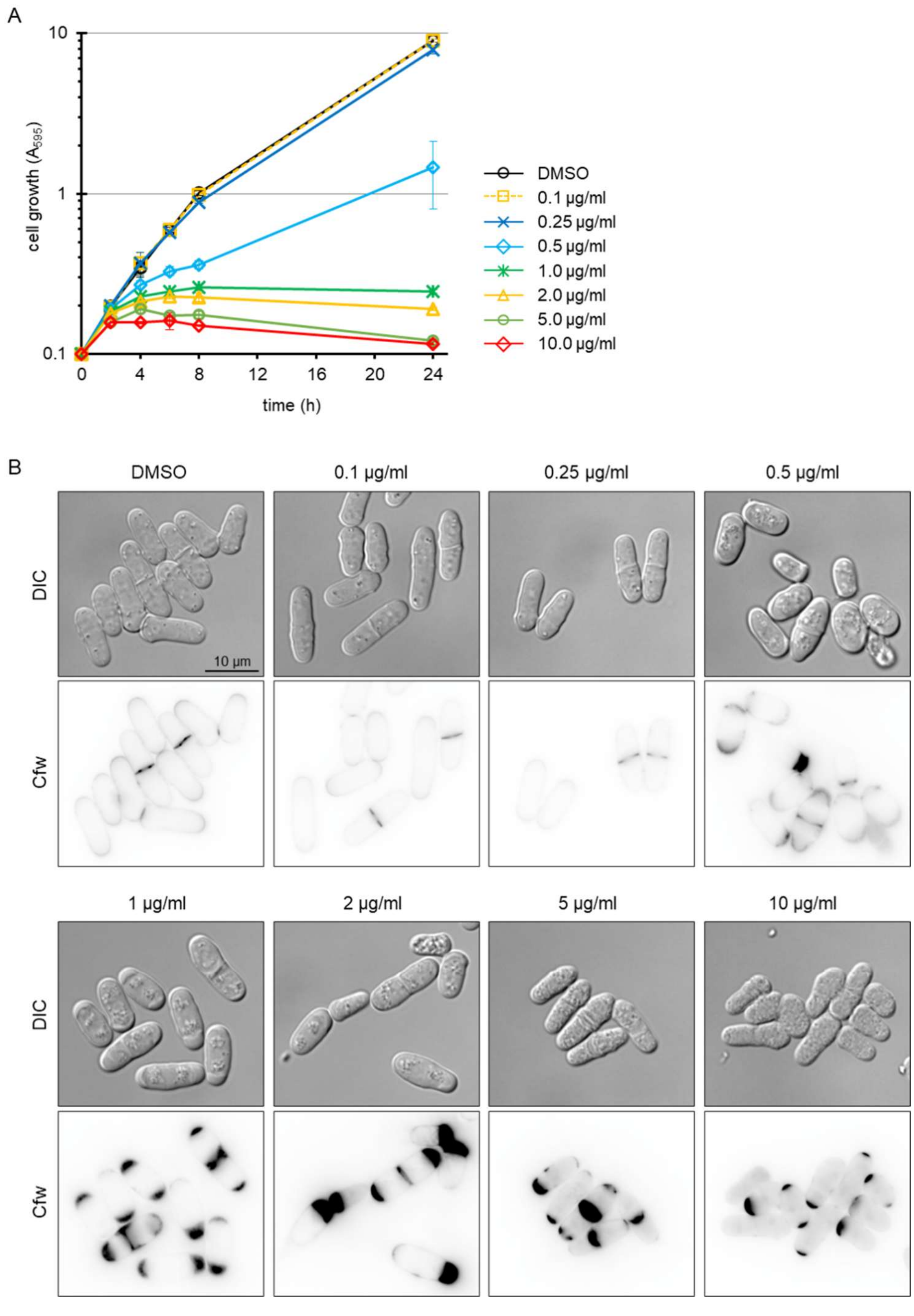

Figure S1. Concentration dependence of the effect of TNM-A, related to Figure 1.

(A) Early log-phase wild-type cells growing in YES medium at 27 °C were treated with TNM-A at various concentrations and the cell growth at A_{600} was monitored for 24 h. Data represent the mean ± SD (n = 3).

(B) Cells were treated for 7 h with increasing concentrations of TNM-A, stained with calcofluor white (Cfw), and observed under Differential Interference Contrast (DIC) and fluorescence microscopy. Scale bar (10 µm) is shown in the control, DIC panel.

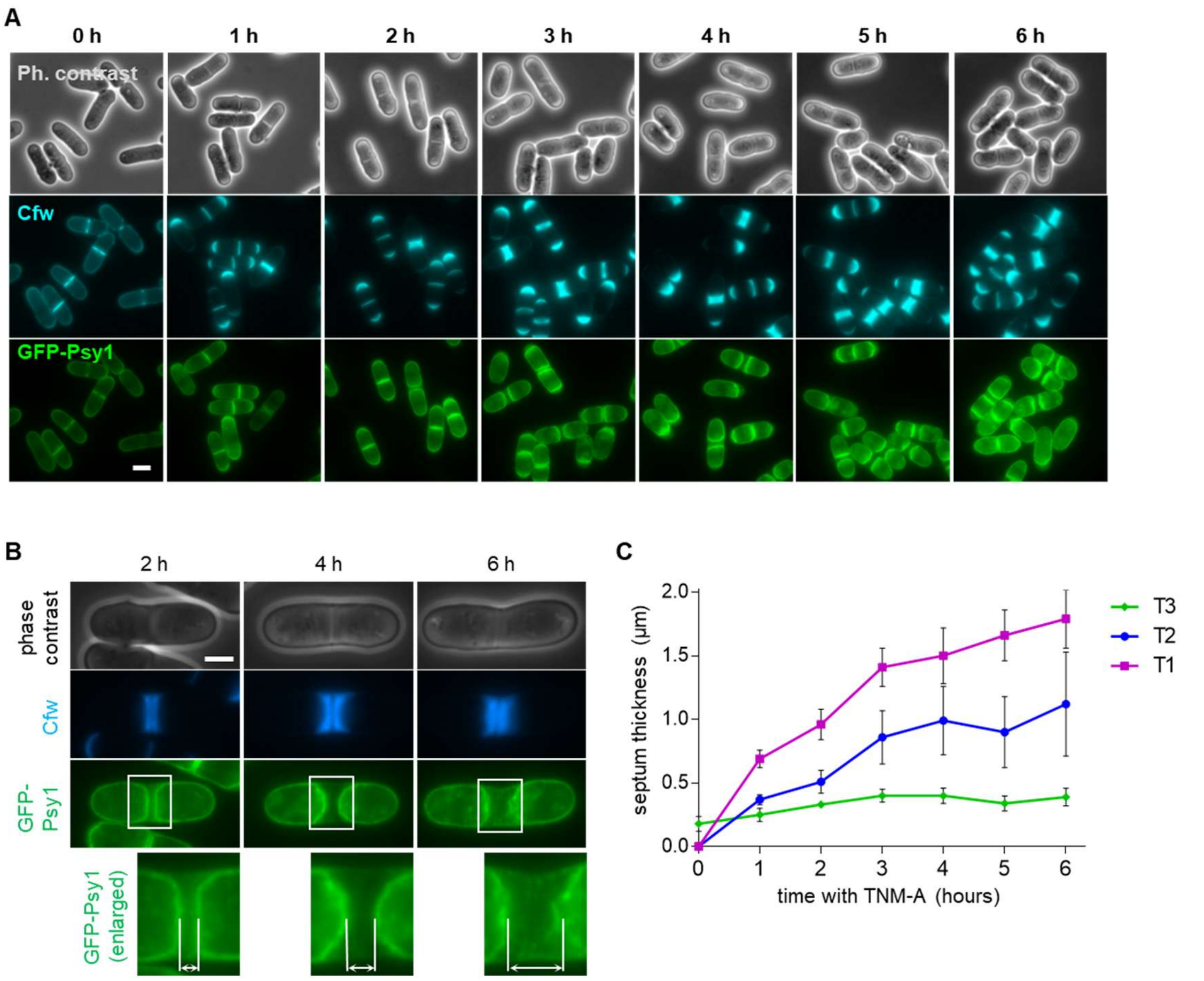

Figure S2. Time course analyses of the morphological changes induced by TNM-A, related to Figure 1.

(A) Cells with GFP-Psy1 were incubated in YES medium containing 2.0 μg/mL of TNM-A under shaking at 28 °C for 6 h. At each hour, cells were collected and analyzed by phase contrast and Cfw and GFP-Psy1 fluorescence microscopy.

(B) Cells with the T1 phenotype, as in Figure 1B and C, at 2 h, 4 h and 6 h of TNM-A treatment. The septum areas with boxes are enlarged in the bottom panels and the distance between the opposing membranes surrounding the septum was measured. Scale bars, 5 μm (A), and 2.5 μm (B).

(C) Septum thickness of each of the three types of septation phenotype, T1, T2, and T3 as in Figure 1B and C, in cells growing with TNM-A for 6 h. Data represent the mean ± SD (n = 3).

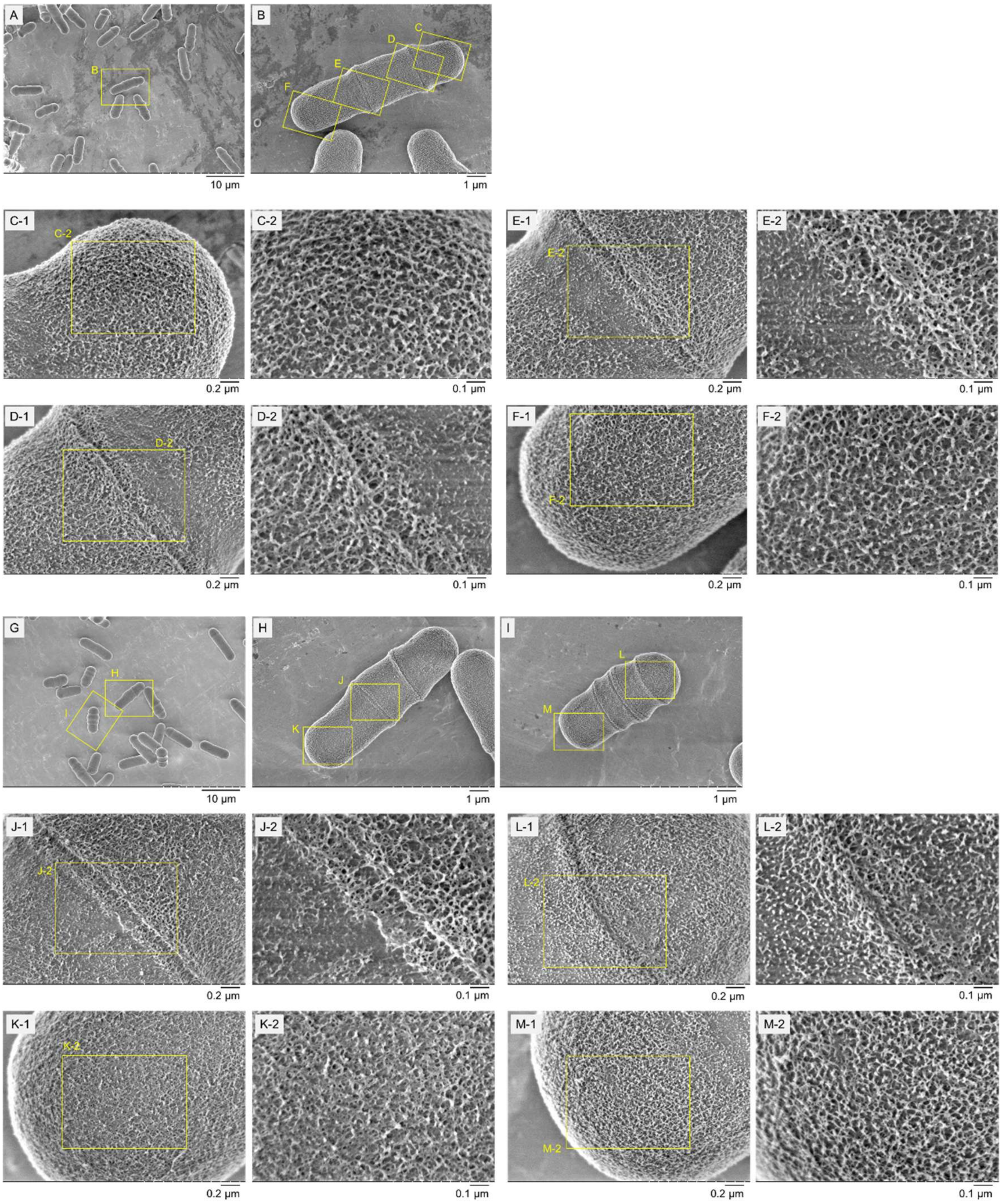

Figure S3. Scanning electron microscopy analyses of the cell wall surface of fission yeast cells treated with TNM-A, related to Figure 1.

(A-F) Scanning electron microscopy (SEM) images of cells treated with DMSO. (J-M) SEM images of cells treated with TNM-A. Cells were treated with DMSO (0.5%) or TNM-A (2 µg/ml) for 2 h at 27 °C. Areas with boxes are marked with a letter and enlarged in the following panel with the same letter. The last areas with boxes of the

most magnified images are enlarged in the right panels. Scale bars are shown for each panel.

Percentage of cell wall in the cell.

	% cell wall/cell ± SD
**Control**	34.15 ± 1.84 (100%)[a]
**TNM-A 2 h**	43.24 ± 8.54 (127%)
**TNM-A 4 h**	54.47 ± 12.75 (160%)
**TNM-A 6 h**	56.11 ± 12.62 (164%)

[a] Percentage of the amount of cell wall in the cell with TNM-A with respect to the control amount with DMSO.

Percentage of the polysaccharides in the cell.

		Polysaccharides (%) [a]			β-glucans (%) [a]	
		α-glucan	β-glucans	Galactomannoproteins	β(1,3)-glucan	β(1,6)-glucan
**DMSO**		9.77 ± 1.13 (100%)[b]	19.43 ± 2.34 (100%)[b]	4.95 ± 1.4 (100%)[b]	18.31 ± 2.22 (100%)[b]	1.12 ± 3.13 (100%)[b]
**TNM-A**	**2 h**	14.53 ± 0.36 (149%)	26.96 ± 0.92 (139%)	1.75 ± 0.89 (35%)	26.21 ± 0.68 (143%)	0.75 ± 1.05 (67%)
	**4 h**	15.08 ± 1.63 (154%)	36.41 ± 1.62 (187%)	2.98 ± 0.84 (60%)	36.41 ± 1.62 (199%)	0 (0%)
	**6 h**	12.65 ± 0.54 (129%)	39.62 ± 1.15 (204%)	3.84 ± 0.93 (78%)	39.62 ± 1.15 (216%)	0 (0%)

[a] Percentage of each polysaccharide in the cell.
[b] Percentage of the amount of each polysaccharide in the cell with TNM-A with respect to the control amount with DMSO.

Percentage of the polysaccharides in the cell wall.

		Polysaccharides (%) [a]			β-glucans (%) [a]	
		α-glucan	β-glucans	Galactomannoproteins	β(1,3)-glucan	β(1,6)-glucan
**DMSO**		28.60 ± 1.13 (100%) [b]	56.90 ± 2.34 (100%) [b]	14.50 ± 1.4 (100%) [b]	53.63 ± 2.22 (100%) [b]	3.28 ± 3.13 (100%) [b]
**TNM-A**	**2 h**	33.60 ± 0.36 (117%)	62.34 ± 0.92 (110%)	4.05 ± 0.89 (28%)	60.61 ± 0.68 (113%)	1.74 ± 1.05 (53%)
	**4 h**	27.69 ± 1.63 (97%)	66.84 ± 1.62 (117%)	5.47 ± 0.84 (38%)	66.84 ± 1.62 (125%)	0 (0%)
	**6 h**	22.55 ± 0.54 (79%)	70.61 ± 1.15 (124%)	6.85 ± 0.93 (47%)	70.61 ± 1.15 (132%)	0 (0%)

[a] Percentage of each polysaccharide in the cell wall.
[b] Percentage of the amount of each polysaccharide in the cell wall with TNM-A with respect to the control amount with DMSO.

Figure S4. The effect of TNM-A on the percentage of the cell wall in the cell, and on the percentage of the polysaccharides in the cell, related to Figure 2.

Bar graphs for these data are shown in Figure 2. Data represent the mean ± SD (n = 4).

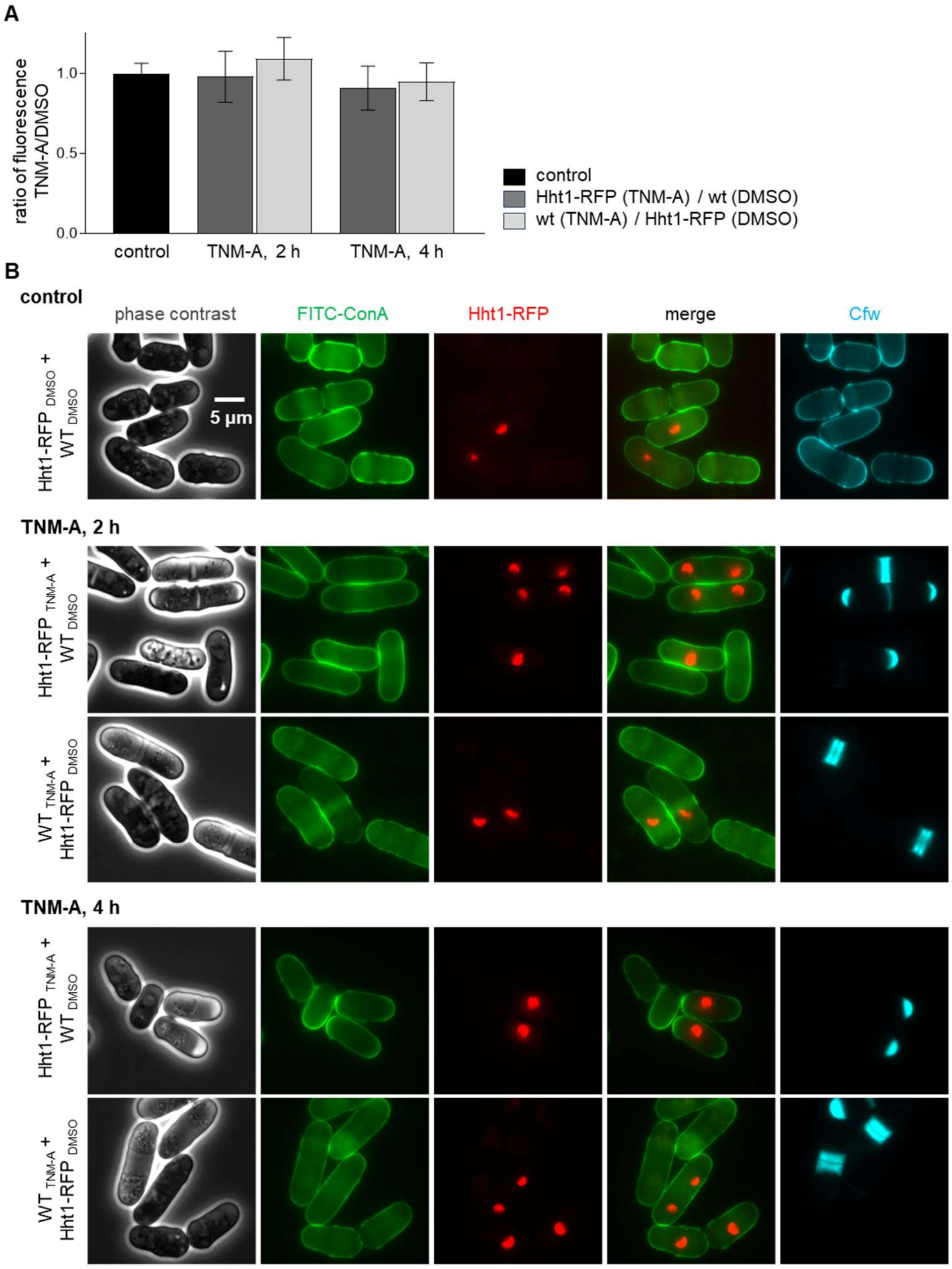

Figure S5. Effect of TNM-A on the amount of galactomannoproteins of the outer surface of the cell wall, related to Figure 2.

(A) Quantification of the amount of galactomannan on the cell surface. Bars represent the mean ± SD (n = 4) of the rate of the maximum fluorescence of the ROI on the surface of two compared strains with and without Hht1-RFP to allow their discrimination. 819 cells with TNM-A and 1650 cells with DMSO were measured (≧ 406 cells in each condition). No statistically significant difference was observed.

(B) Images of the cell mixture. Early log-phase wild-type (WT) cells were treated with TNM-A, and the Hht1-RFP strain was treated with DMSO, and the opposite WT with DMSO and the Hht1-RFP strain with TNM-A, which were mixed and mounted. Scale bar (5 μm) is shown in the control, phase contrast panel.

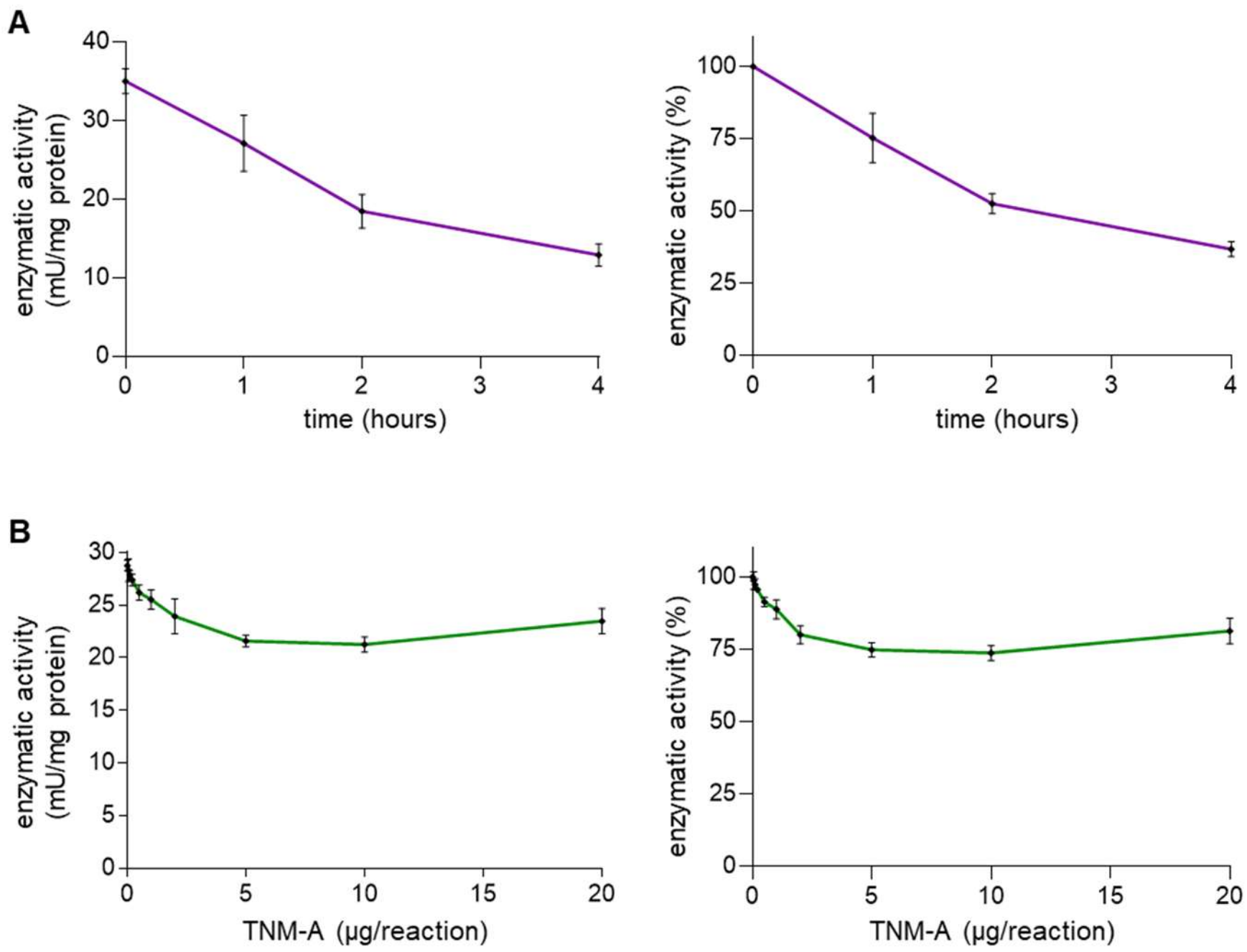

Figure S6. The effect of TNM-A on the *in vivo* and *in vitro* 1,3-ß-glucan synthase activities, related to Figure 2.

(A) The effect of TNM-A on the cellular (*in vivo*) activity of 1,3-ß-glucan synthase (GS). Early log-phase cells were cultured at 28 °C in YES with 2 µg/mL TNM-A for 0, 1, 2, and 4 hours. All cell cultures were harvested at 1.0 A_{600} ($1x10^7$ cells/mL) and their membranes were extracted to quantify the GS activity.

(B) The effect of TNM-A on the *in vitro* activity of GS. Early log-phase cells were cultured at 28 °C in YES, and cell cultures were harvested at 1.0 A_{600} ($1x10^7$ cells/mL) to prepare a cell membrane fraction that contains the enzyme complex. TNM-A was added to the reaction mixture to assess the effect of the compound on the GS activity.

Left graphs: specific activity is expressed as milliunits per milligram of protein (mU/mg). Right graphs: percentage values of specific activity compared to control activity in the absence of TNM-A. Data represent the mean ± SD (n = 4).

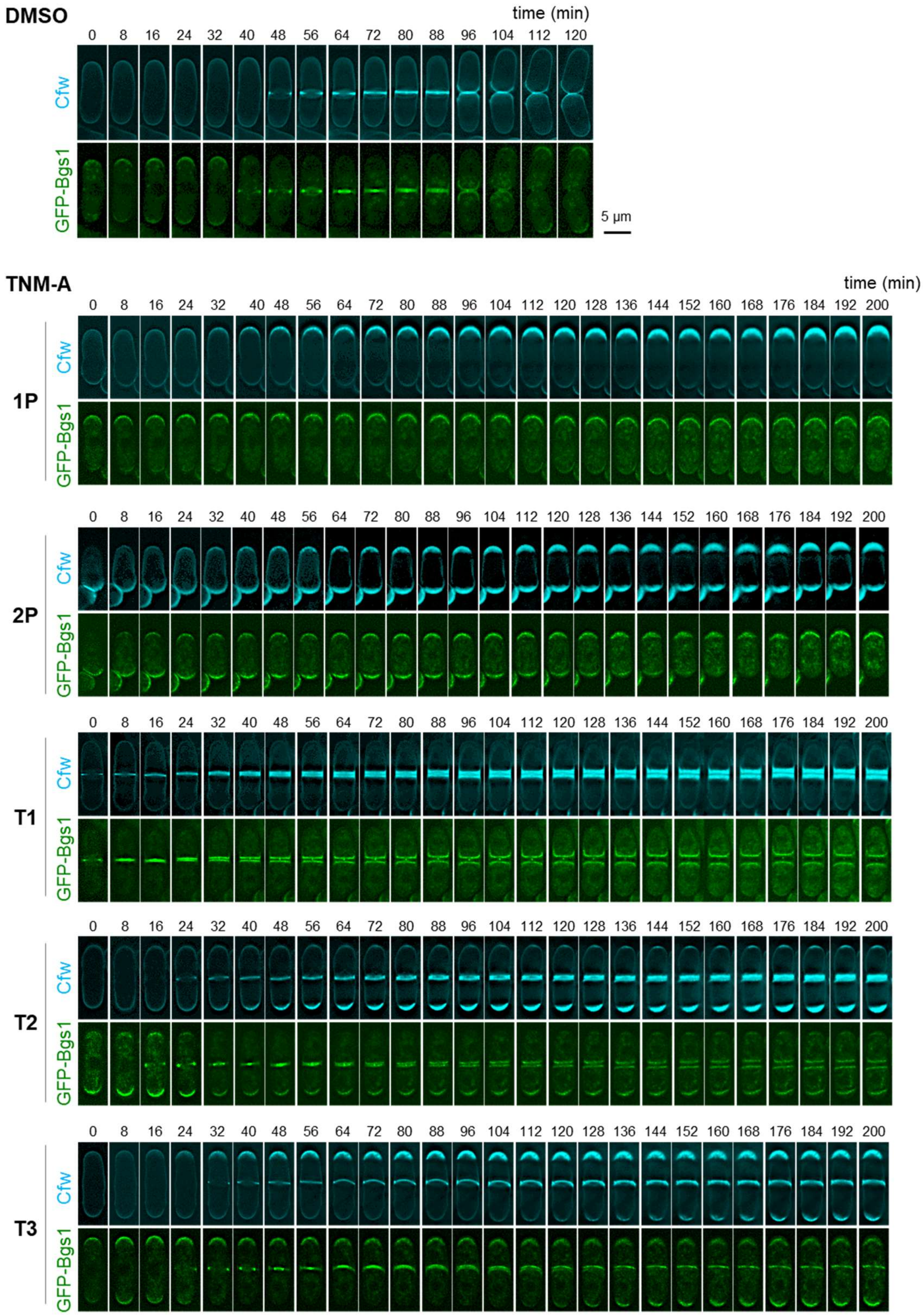

Figure S7. Time lapse images of GFP-Bgs1 in the presence of TNM-A, related to Figure 3. Time lapse images of cells containing GFP-Bgs1. Cells were incubated in YES medium at 28 °C with DMSO (control) or TNM-A (2.0 μg/mL). Image capture was performed

every 8 minutes for at least 120 minutes in different channels to observe Cfw staining (top, cyan) and GFP (bottom, green) fluorescence. Control cells and TNM-A treated cells with 1P, 2P, T1, T2, and T3 phenotypes, as in Figure 1B and C, are shown. Control, 1P and T2 cells are also shown in Figure 3B, and T1 cells in Figure S2B. Scale bar (5 μm) is shown next to the control DMSO treatment panels.

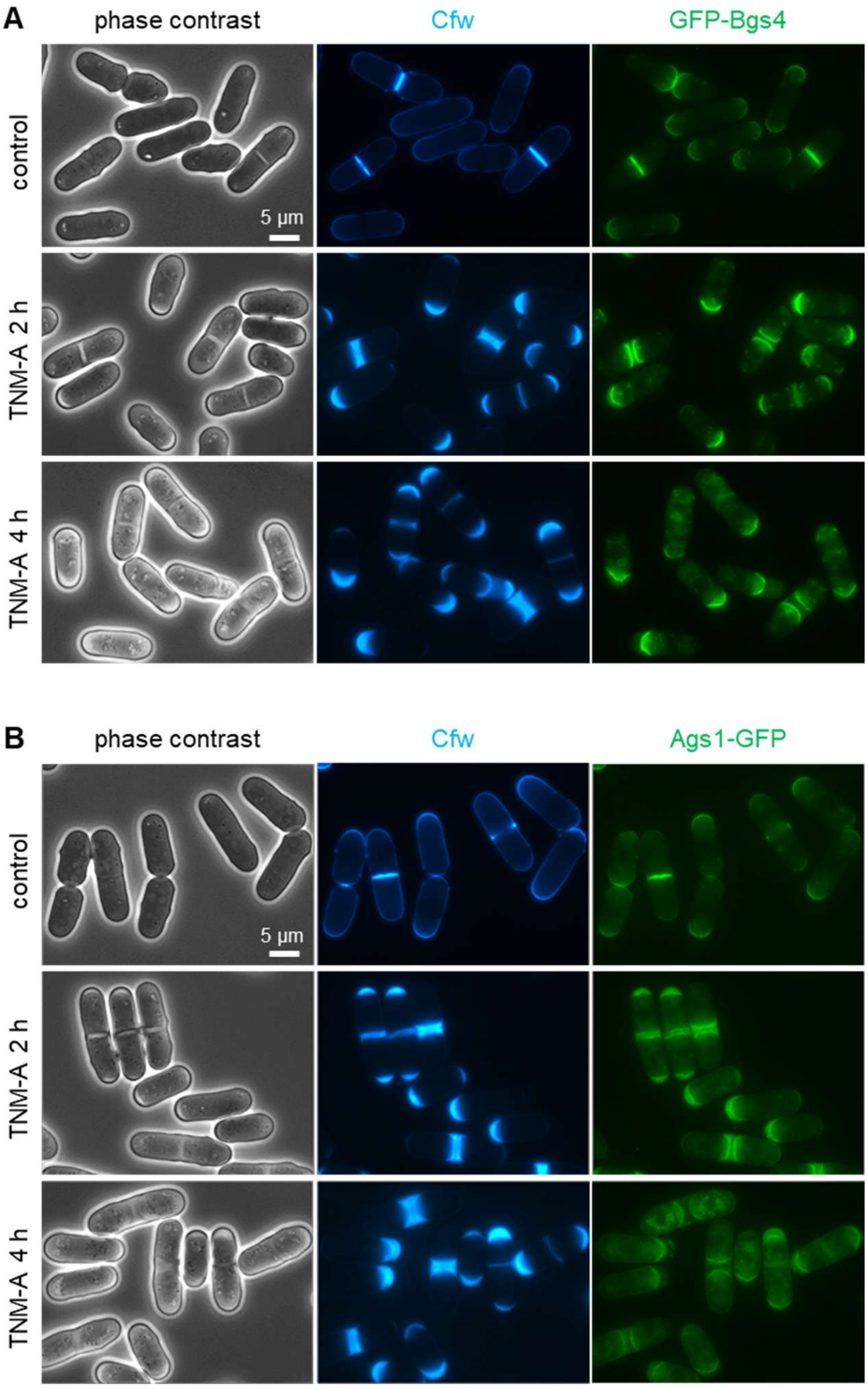

Figure S8. Forced permanent localization of GFP-Bgs4 and Ags1-GFP by TNM-A, related to Figure 3.

Localization of GFP-Bgs4 (A), or Ags1-GFP (B) in the presence of TNM-A. Cells expressing GFP-Bgs4, or Ags1-GFP were treated with TNM-A (2ug/mL). Images of phase contrast, and Cfw (mainly linear 1,3-ß-glucan), and GS (GFP-Bgs4 in (A), Ags1-GFP in (B)) fluorescence are shown. Scale bars (5 µm) are shown in the control, phase contrast panels.

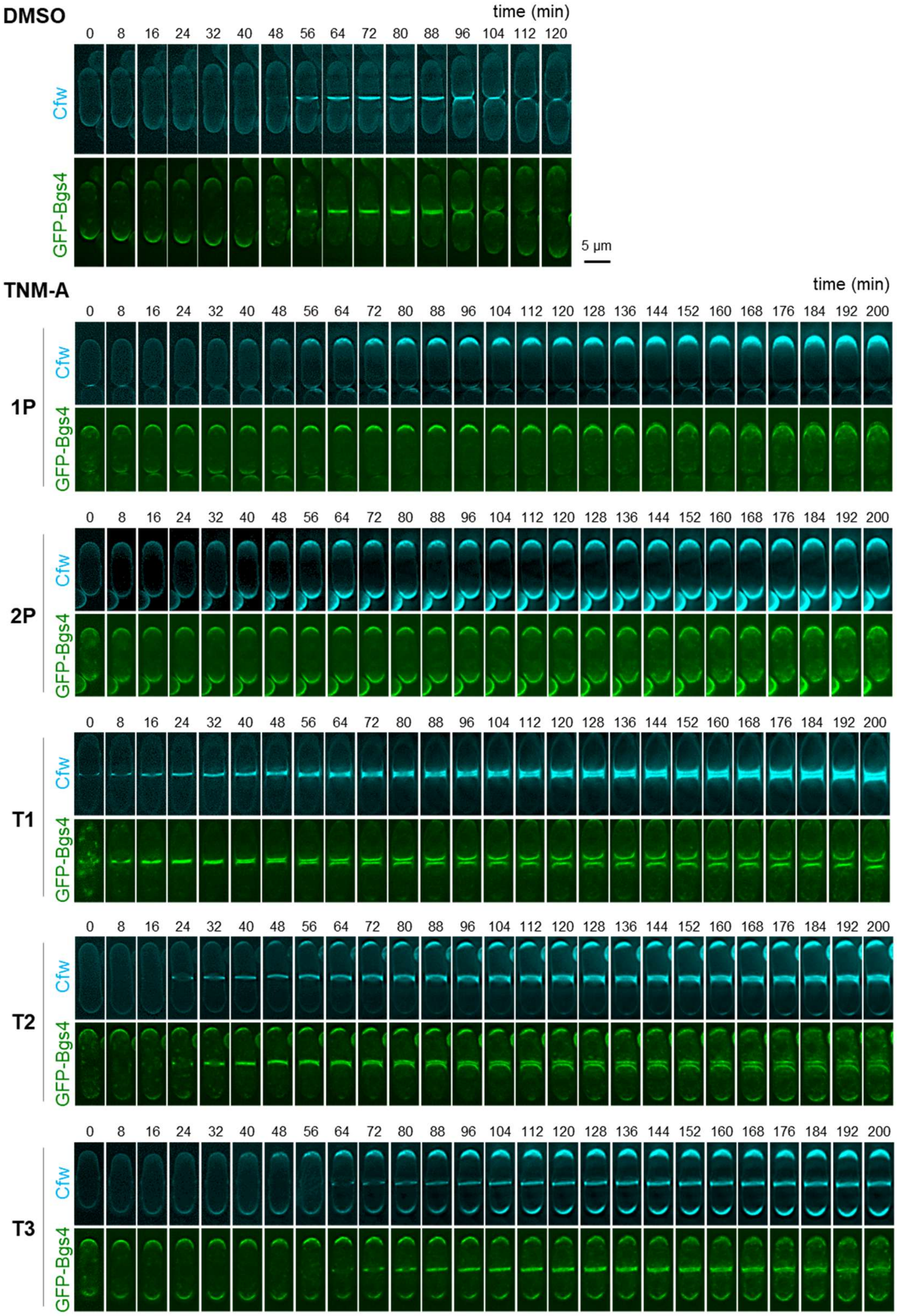

Figure S9. Time lapse images of GFP-Bgs4 in the presence of TNM-A, related to Figure 3. Time lapse images of cells containing GFP-Bgs4. Cells were incubated in YES medium at 28 °C with DMSO (control) or TNM-A (2.0 μg/mL). Image capture was performed

every 8 minutes for at least 120 minutes in different channels to observe Cfw staining (top, cyan) and GFP (bottom, green) fluorescence. Control cells and TNM-A treated cells with 1P, 2P, T1, T2, and T3 phenotypes, as in Figure 1B and C, are shown. Control, 1P and T2 cells are also shown in Figure 3B, and T1 cells in Figure S2B. Scale bar (5 μm) is shown next to the control DMSO treatment panels.

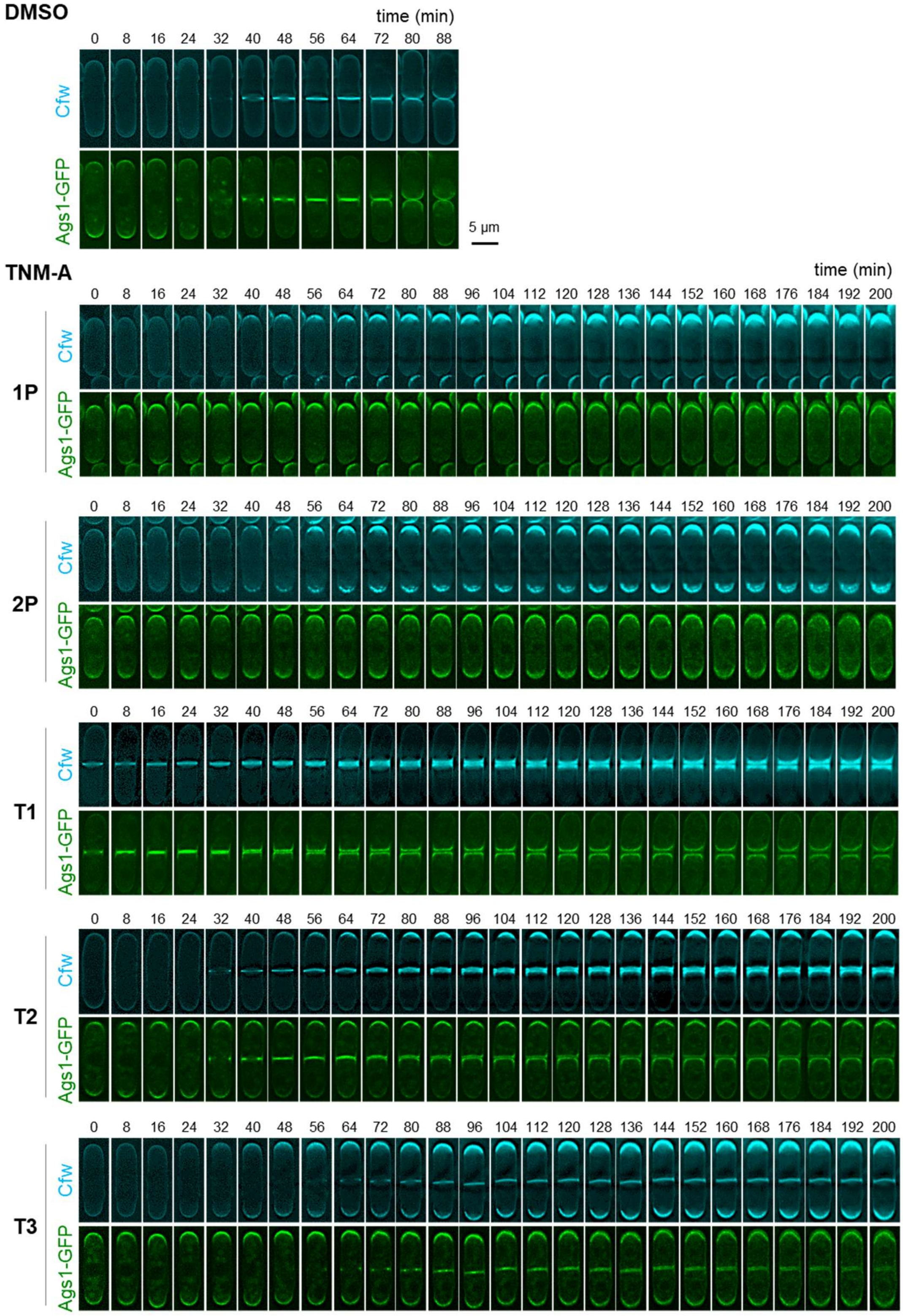

Figure S10. Time lapse images of Ags1-GFP in the presence of TNM-A, related to Figure 3. Time lapse images of cells containing Ags1-GFP. Cells were incubated in YES medium at 28 °C with DMSO (control) or TNM-A (2.0 μg/mL). Image capture was performed

every 8 minutes for at least 90 minutes in different channels to observe Cfw staining (top, cyan) and GFP (bottom, green) fluorescence. Control cells and TNM-A treated cells with 1P, 2P, T1, T2, and T3 phenotypes, as in Figure 1B and C, are shown. Control, 1P and T2 cells are also shown in Figure 3B, and T1 cells in Figure S2B. Scale bar (5 μm) is shown next to the control DMSO treatment panels.

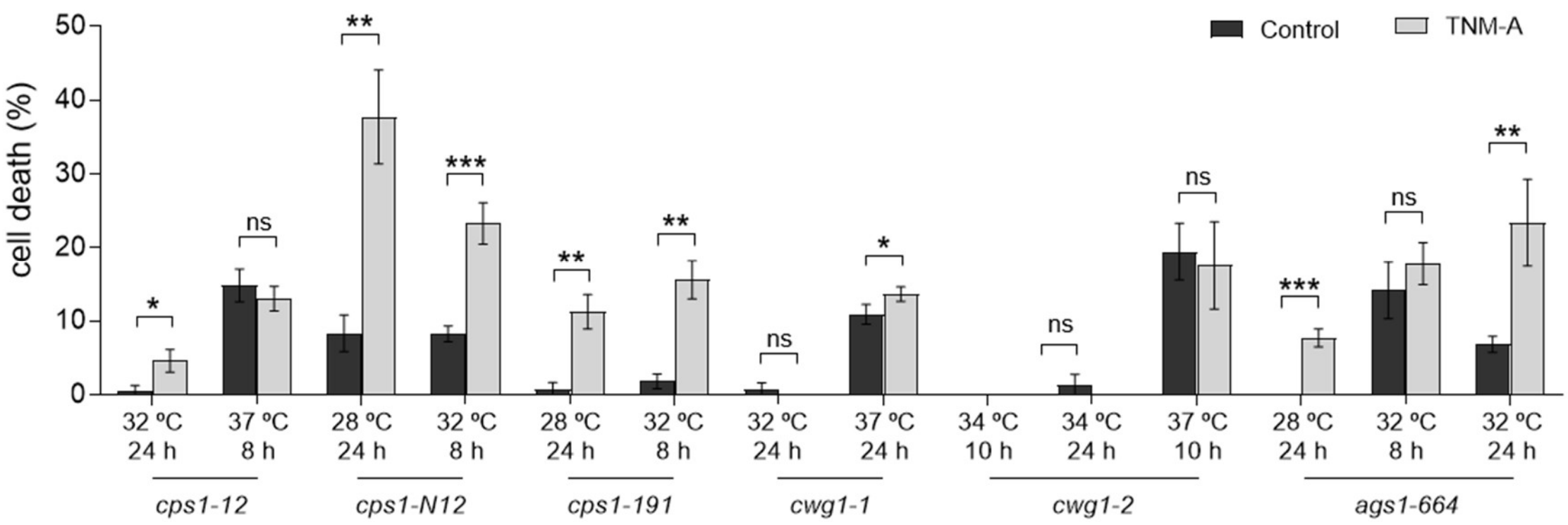

Figure S11. Effect of TNM-A on the survival of GS mutant strains, related to Figure 3. GS mutant strains were grown in YES at 25 °C and were maintained in log phase. Then, each mutant was transferred to a semi-permissive and restrictive growth condition. The time indicates the maximum period each mutant can sustain at this temperature before growth stops. The last 4 hours of growth in semi-permissive or restrictive condition, TNM-A or DMSO (control) was added to the cultures. The wild-type strain is not shown, since it did not show cell death in any conditions. Data represent the mean ± SD (n = 3; ≧ 142 cells in each condition, total 13097 cells analyzed). The asterisks indicate the significant statistical difference between paired strains analyzed by the Student's t-test: *p < 0.05; **p < 0.01; ***p < 0.001; ns: not significant (p > 0.05).

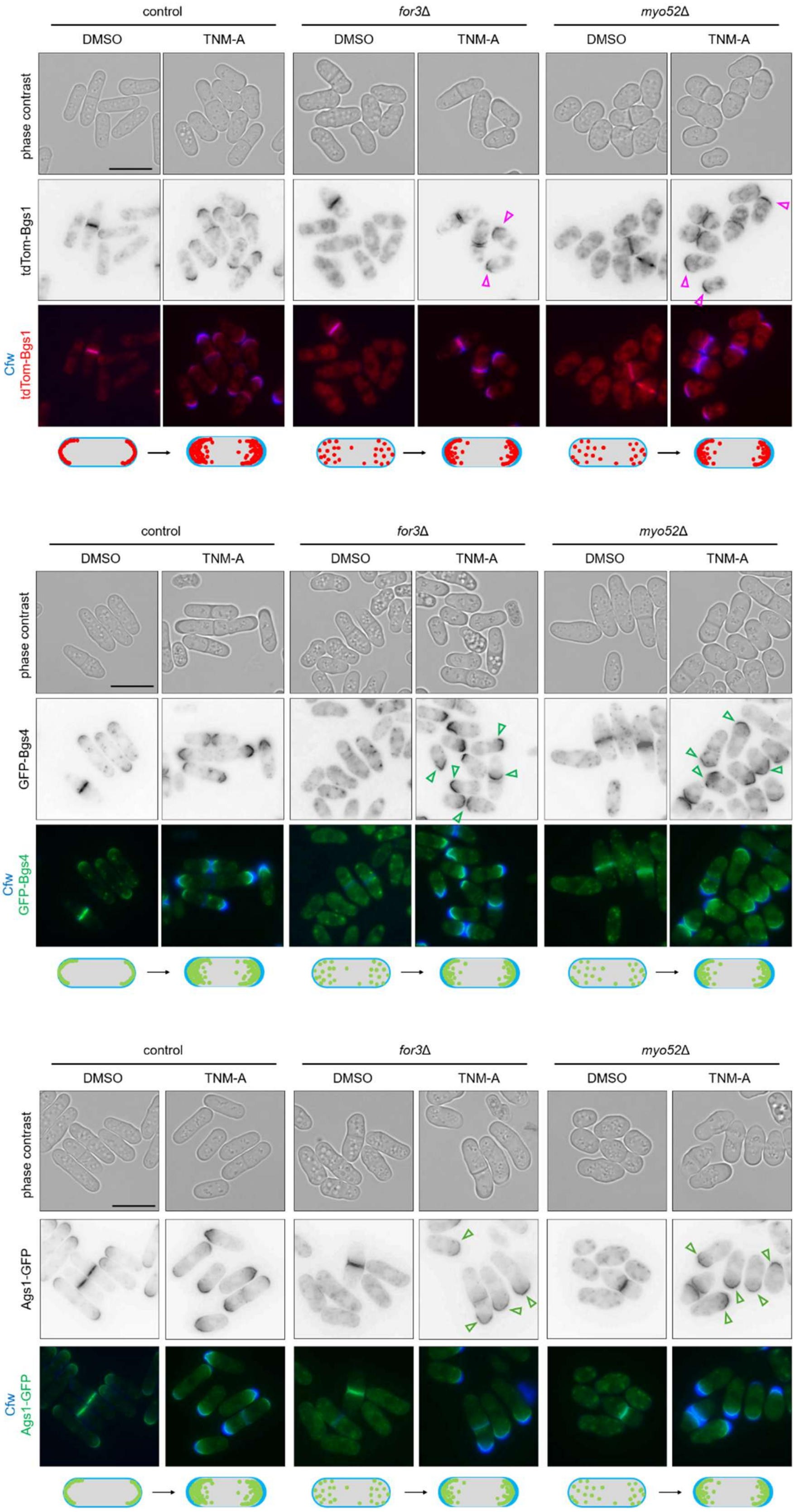

Figure S12. Effect of *for3* or *myo52* deletion on the localization of Bgs1, Bgs4, and Ags1,

related to Figure 6.

Control, *for3*Δ, or *myo52*Δ cells containing tdTom-Bgs1, GFP-Bgs4, or Ags1-GFP were treated with DMSO (control) or TNM-A (2 µg/ml) for 2 h. Cells were stained by Cfw (mainly linear 1,3-ß-glucan) and observed under phase contrast and Cfw, tdTom, and GFP fluorescence microscopy. Scale bars (10 µm) are shown in the upper left, brightfield images. Open triangle; accumulation of tdTom-Bgs1 (red triangle), GFP-Bgs4, and Ags1-GFP (green triangle) at the poles. A scheme of the accumulation of Cfw, and tdTom-Bgs1, GFP-Bgs4, or Ags1-GFP at the poles with TNM-A is depicted bellow each series. Some of the pictures also appear in Figure 6.

Table S1. List of 32 proteins that are localized at sites with thick cell wall in cells treated with TNM-A, related to Figure 4

Gene Systematic ID	Gene standard name; Synonyms	Gene Product
SPAP8A3.08	*cdc4*	myosin II essential light chain
SPBC1198.03c		DUF4646 family conserved fungal protein
SPAC17G8.10c	*dma1*	mitotic spindle checkpoint ubiquitin ligase Dma1
SPBC83.18c	*fic1*	contractile ring-membrane anchor Fic1
SPAC5D6.09c	*mug86*	plasma membrane acetate transmembrane transporter
SPBC6B1.03c	*pal2*	pal1 family early clathrin mediated endocytosis factor, meiosis specific
SPCC1739.10	*mug33*	Tea1-interacting protein implicated in exocytosis
SPCC1753.02c	*git3*	G-protein coupled receptor Git3
SPAC637.13c	*slm1*	cytoskeletal signaling protein Slm1
SPAC23C4.10	*sec2*	Rab GEF Sec2
SPBC947.01	*knk1*; *alf1*	AAA family ATPase kink, Knk1
SPBC23G7.08c	*rga7*	RhoGAP Rga7
SPBP8B7.27	*mug30*	HECT-type ubiquitin-protein ligase E3, found in association with TRAMP complex
SPCC1020.01c	*pma2*	P-type proton ATPase, P3-type Pma2
SPAC607.10	*spo3*	sporulation protein Spo3
SPAC20G8.05c	*cdc15*	F-BAR domain protein Cdc15
SPBC27.04	*uds1*	septation protein Uds1
SPCC895.05	*for3*	formin For3
SPCC1919.10c	*myo52*; *myo4*	myosin type V
SPAC22H10.07	*scd2*; *ral3*	Cdc42 GTPase complex scaffold subunit Scd2
SPAC19A8.03	*ymr1*	phosphatidylinositol-3-phosphatase, myotubularin family
SPAC607.06c	*ppp57*	metallopeptidase
SPBC21D10.05c	*ucp3*; *soc2*	ArfGAP, GTPase activating protein for Arf6, Ucp3
SPAC9G1.05	*aip1*	actin binding WD repeat protein Aip1
SPBC16E9.11c	*pub3*	HECT-type ubiquitin-protein ligase E3 Pub3
SPAC11E3.02c	*ync13*	Munc family exocytic/endocytic regulator Ync13
SPBC1604.14c	*shk1; pak1; orb2*	PAK-related kinase Shk1
SPBP8B7.26		Schizosaccharomyces specific protein
SPAC144.14	*klp8*	kinesin-like protein Klp8
SPAPJ760.02c	*abp1*	cofilin/tropomyosin family, drebrin ortholog Abp1
SPAC9G1.10c	*inp53*	inositol polyphosphate phosphatase Inp53
SPBC32H8.13c	*mok12*	prospore membrane alpha-1,3-glucan synthase Mok12

Table S2. *S. pombe* strains used in this work, related to STAR Methods

strain	genotype	origin
JY1	h^-	lab collection
KGY16700	h^+ mCherry-cdc15$^+$:kan^r sid4$^+$-RFP:kanR ade6-M21X leu1-32 ura4-Δ18	K.L. Gould [a]
KN2	h^- mCherry-cdc15$^+$:kan^r	this study
CA5931	h^- leu1-32 ura4-294::[shk1 promoter:ScGIC2 CRIB-GFP3:ura4$^+$]	K. Shiozaki [b]
JCR3591	h^+ leu1-32 ura4-Δ18 his3-Δ1 bgs1Δ::ura4$^+$ Pbgs1$^+$::tdTom-12A-bgs1$^+$:leu1$^+$	lab collection
JCR3168	h^- leu1-32 ura4-Δ18 his3-Δ1 ags1Δ 3'UTRags1$^+$::ags1$^+$-12A-GFP-12A:leu1$^+$:ura4$^+$	lab collection
KN18	h^- ags1Δ 3'UTRags1$^+$::ags1$^+$-12A-GFP-12A: leu1$^+$:ura4$^+$	this study
KN21	h^+ ags1Δ 3'UTRags1$^+$::ags1$^+$-12A-GFP-12A: leu1$^+$:ura4$^+$ for3Δ::kan^r	this study
KN23	h^- ags1Δ 3'UTRags1$^+$::ags1$^+$-12A-GFP-12A: leu1$^+$:ura4$^+$ myo52Δ::kan^r	this study
JCR1780	h^- leu1-32 ura4-Δ18 his3-Δ1 bgs1Δ::ura4$^+$ Pbgs1$^+$::tdTom-12A-bgs1$^+$:leu1$^+$	lab collection
KN24	h^- bgs1Δ::ura4$^+$ Pbgs1$^+$::tdTom-12A-bgs1$^+$:leu1$^+$	this study
KN26	h^- bgs1Δ::ura4$^+$ Pbgs1$^+$::tdTom-12A-bgs1$^+$:leu1$^+$ for3Δ::kan^r	this study
KN28	h^{90} bgs1Δ::ura4$^+$ Pbgs1$^+$::tdTom-12A-bgs1$^+$:leu1$^+$ myo52Δ::kan^r	this study
KN36	h^- bgs4Δ::ura4$^+$ Pbgs4$^+$::GFP-12A-bgs4$^+$:leu1$^+$	this study
KN38	h^+ bgs4Δ::ura4$^+$ Pbgs4$^+$::GFP-12A-bgs4$^+$:leu1$^+$ for3Δ::kan^r	this study
KN41	h^- bgs4Δ::ura4$^+$ Pbgs4$^+$::GFP-12A-bgs4$^+$:leu1$^+$ myo52Δ::kan^r	this study
JCR33	h^- 972	P. Munz [c]
JCR77	h^- leu1-32	lab collection
JCR284	h^- leu1-32 ura4-Δ18 his3-Δ1	lab collection
JCR420	h^+ leu1-32 ura4-Δ18	lab collection
JCR1722	h^- leu1-32 ura4-Δ18 his3-Δ1 bgs1Δ::ura4$^+$ Pbgs1$^+$::GFP-12A-bgs1$^+$:leu1$^+$	lab collection
JCR1723	h^+ leu1-32 ura4-Δ18 his3-Δ1 bgs1Δ::ura4$^+$ Pbgs1$^+$::GFP-12A-bgs1$^+$:leu1$^+$	lab collection
JCR2364	h^- leu1-32 ura4-Δ18 his3-Δ1 bgs4Δ::ura4$^+$ Pbgs4$^+$::GFP-12A-bgs4$^+$:leu1$^+$	lab collection
JCR2365	h^+ leu1-32 ura4-Δ18 his3-Δ1 bgs4Δ::ura4$^+$ Pbgs4$^+$::GFP-12A-bgs4$^+$:leu1$^+$	lab collection
JCR3166	h^- leu1-32 ura4-Δ18 his3-Δ1 ade6-M210 ags1Δ 3'UTRags1$^+$::ags1$^+$-12A-GFP-12A:leu1$^+$:ura4$^+$	lab collection
JCR3167	h^+ leu1-32 ura4-Δ18 his3-Δ1 ade6-M210 ags1Δ 3'UTRags1$^+$::ags1$^+$-12A-GFP-12A:leu1$^+$:ura4$^+$	lab collection
JCR4047	h^- leu1-32 ura4-Δ18 his3-Δ1 ade6-M210 hht1$^+$-RFP:KanMX6	J. Cooper [d]
JCR4079	h^- leu1-32 ura4-Δ18 his3-Δ1 bgs1Δ::ura4$^+$ Pbgs1$^+$::GFP-12A-bgs1$^+$:leu1$^+$ hht1$^+$-RFP:KanMX6	this study
JCR4080	h^- leu1-32 ura4-Δ18 his3-Δ1 bgs4Δ::ura4$^+$ Pbgs4$^+$::GFP-12A-bgs4$^+$:leu1$^+$ hht1$^+$-RFP:KanMX6	this study
JCR4082	h^+ leu1-32 ura4-Δ18 his3-Δ1 ade6-M210 ags1Δ 3'UTRags1$^+$::ags1$^+$-12A-GFP-12A:leu1$^+$:ura4$^+$ hht1$^+$-RFP:KanMX6	lab collection
JCR5016	h^{90} leu1-32 GFP-psy1$^+$:leu1$^+$	C. Shimoda [e]
JCR5062	h^+ leu1-32 GFP-psy1$^+$:leu1$^+$ rlc1$^+$-tdTom:NatMX6	this study
JCR5654	h^- leu1-32 ura4-Δ18 hht1$^+$-RFP:KanMX6	this study
JCR217	h^- leu1-32 cps1-12	lab collection
JCR2865	h^- leu1-32 ura4-Δ18 cps1-N12	V. Simanis [f]
JCR1022	h^- leu1-32 cps1-191	M. Balasubramanian [g]
JCR132	h^- leu1-32 cwg1-1	lab collection
JCR156	h^- leu1-32 cwg1-2	lab collection
JCR4781	h^- leu1-32 ura4-Δ18 ags1-664 (mok1-664)	P. Pérez [h]
a.	Howard Hughes Medical Institute and Department of Cell and Developmental Biology, Vanderbilt University School of Medicine, Nashville, TN.	
b.	Division of Biological Science, Nara Institute of Science and Technology, Ikoma, Japan.	
c.	Institute of Cell Biology, University of Bern, Switzerland	
d.	Department of Biochemistry and Molecular Genetics, University of Colorado Anschutz Medical Campus, Aurora, USA.	
e.	Department of Biology, Graduate School of Science, Osaka City University, Osaka, Japan.	
f.	School of Life Sciences, École Polytechnique Fédérale de Lausanne, Lausanne, Switzerland.	
g.	Centre for Mechanochemical Cell Biology and Division of Biomedical Sciences, Warwick Medical School, Coventry, UK.	
h.	Instituto de Biología Funcional y Genómica, Consejo Superior de Investigaciones Científicas/Universidad de Salamanca, Salamanca, Spain.	
