# Supplementary material for: Ectopic overproduction of cell wall glucan through membrane perturbation by an antifungal peptide theonellamide A in fission yeast
Source: iScience. 2026 Oct 1;29(10):117645. doi: 10.1016/j.isci.2026.117645 (PMC13657213; doi:10.1016/j.isci.2026.117645)
Supplement: Supplementary file 2 [file mmc2.pdf]

A-1

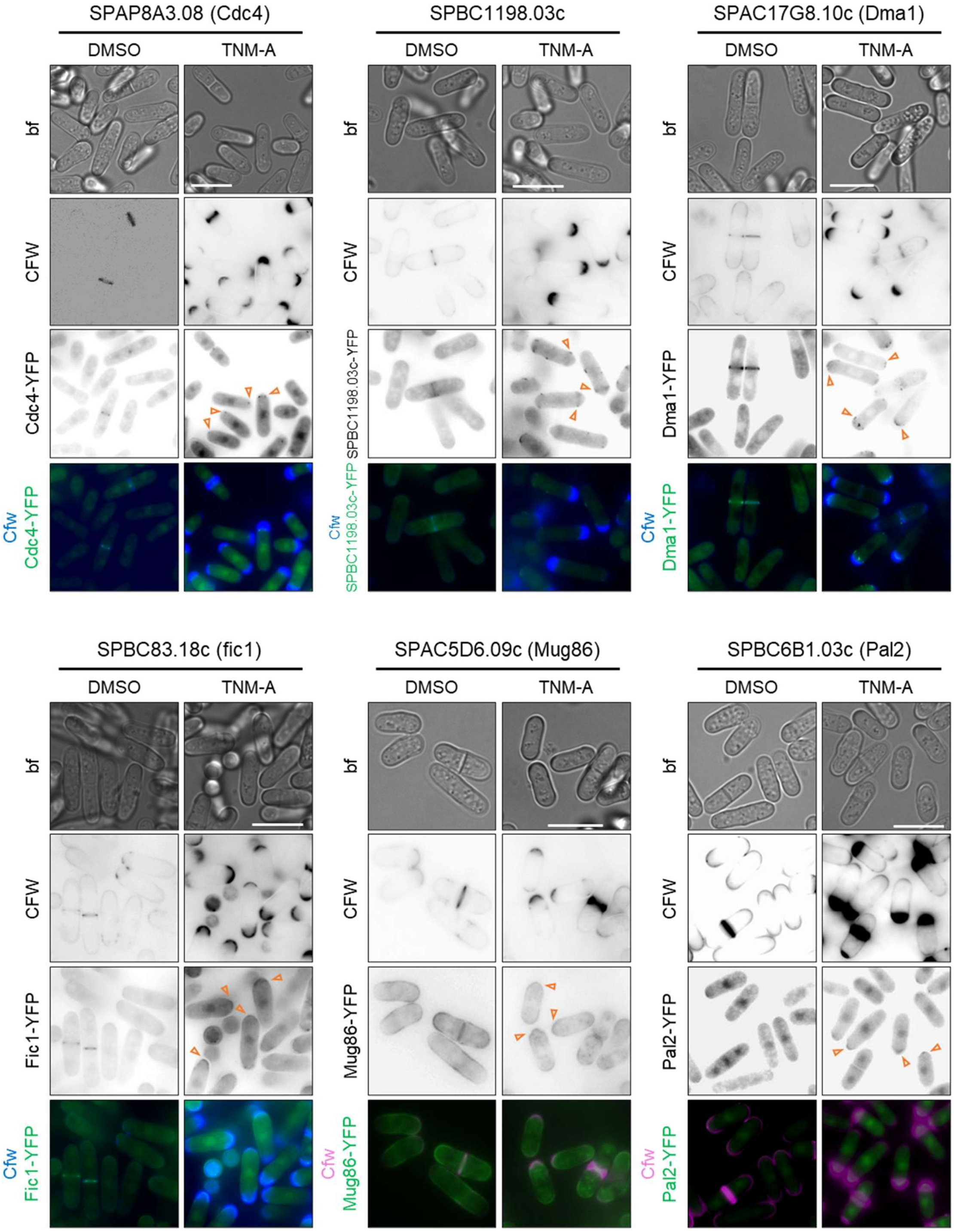
SPAP8A3.08 (Cdc4)
SPBC1198.03c
SPAC17G8.10c (Dma1)
DMSO
TNM-A
bf
CFW
Cdc4-YFP
Cfw
Cdc4-YFP
SPBC1198.03c-YFP
Cfw
SPBC1198.03c-YFP
Dma1-YFP
Cfw
Dma1-YFP
SPBC83.18c (fic1)
SPAC5D6.09c (Mug86)
SPBC6B1.03c (Pal2)
Fic1-YFP
Cfw
Fic1-YFP
Mug86-YFP
Cfw
Mug86-YFP
Pal2-YFP
Cfw
Pal2-YFP

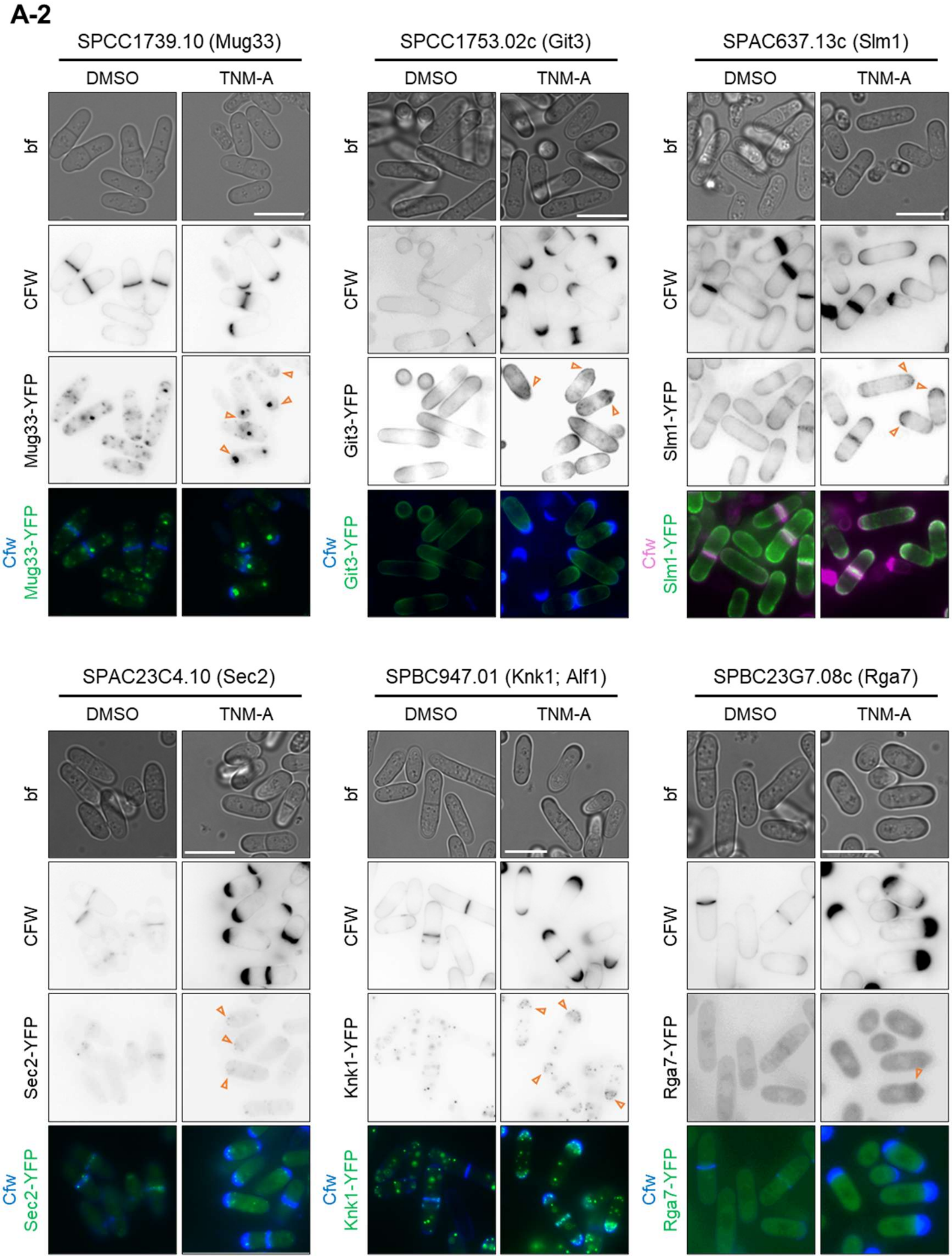

A-2
SPCC1739.10 (Mug33)
SPCC1753.02c (Git3)
SPAC637.13c (Slm1)
DMSO
TNM-A
bf
CFW
Mug33-YFP
Cfw
Mug33-YFP
Git3-YFP
Git3-YFP
Slm1-YFP
Slm1-YFP
SPAC23C4.10 (Sec2)
SPBC947.01 (Knk1; Alf1)
SPBC23G7.08c (Rga7)
Sec2-YFP
Knk1-YFP
Rga7-YFP

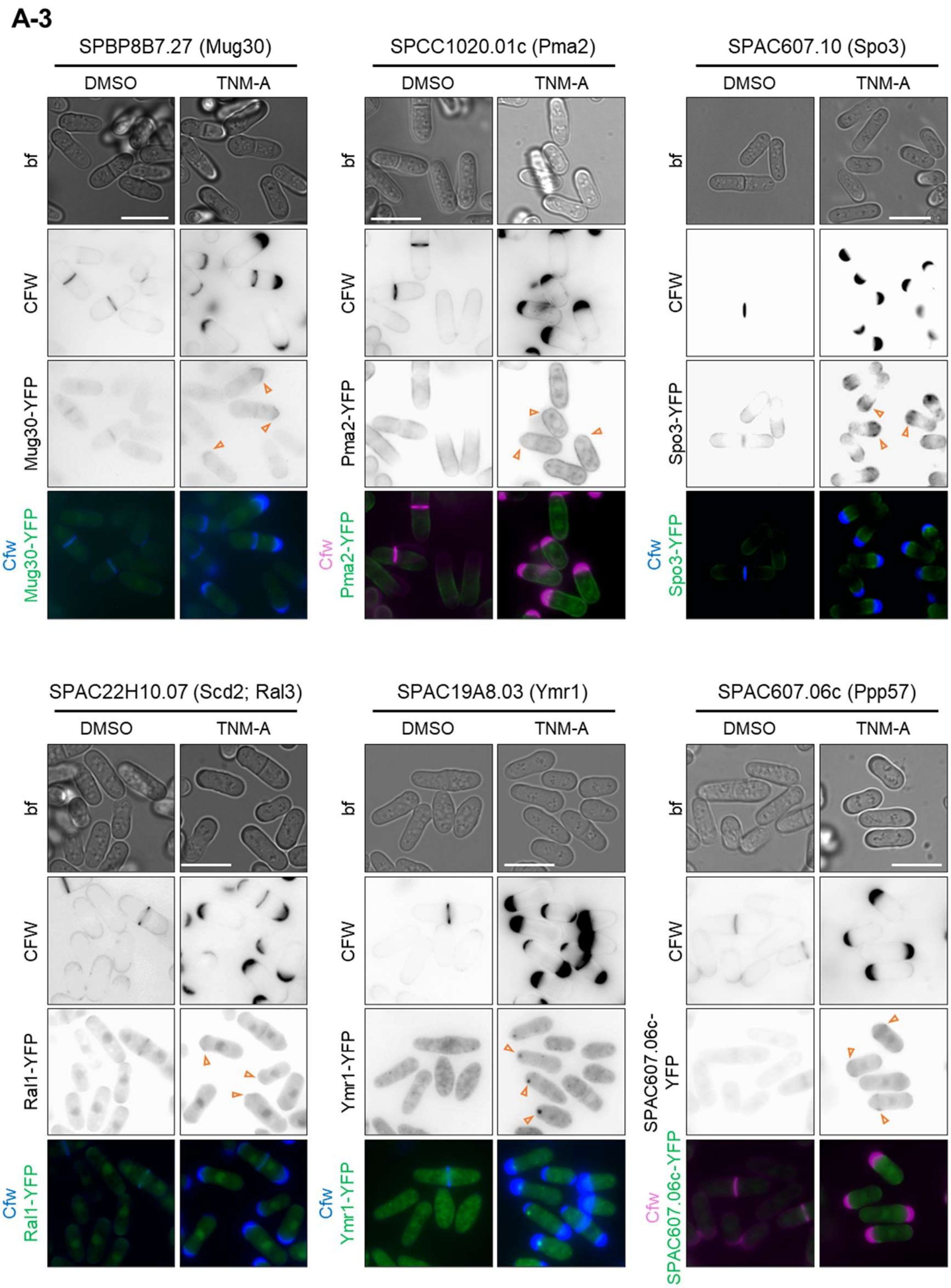
A-3
SPBP8B7.27 (Mug30)
SPCC1020.01c (Pma2)
SPAC607.10 (Spo3)
DMSO
TNM-A
bf
CFW
Mug30-YFP
Cfw
Mug30-YFP
Pma2-YFP
Pma2-YFP
Spo3-YFP
Spo3-YFP
SPAC22H10.07 (Scd2; Ral3)
SPAC19A8.03 (Ymr1)
SPAC607.06c (Ppp57)
Ral1-YFP
Ral1-YFP
Ymr1-YFP
Ymr1-YFP
SPAC607.06c-YFP
SPAC607.06c-YFP

A-4

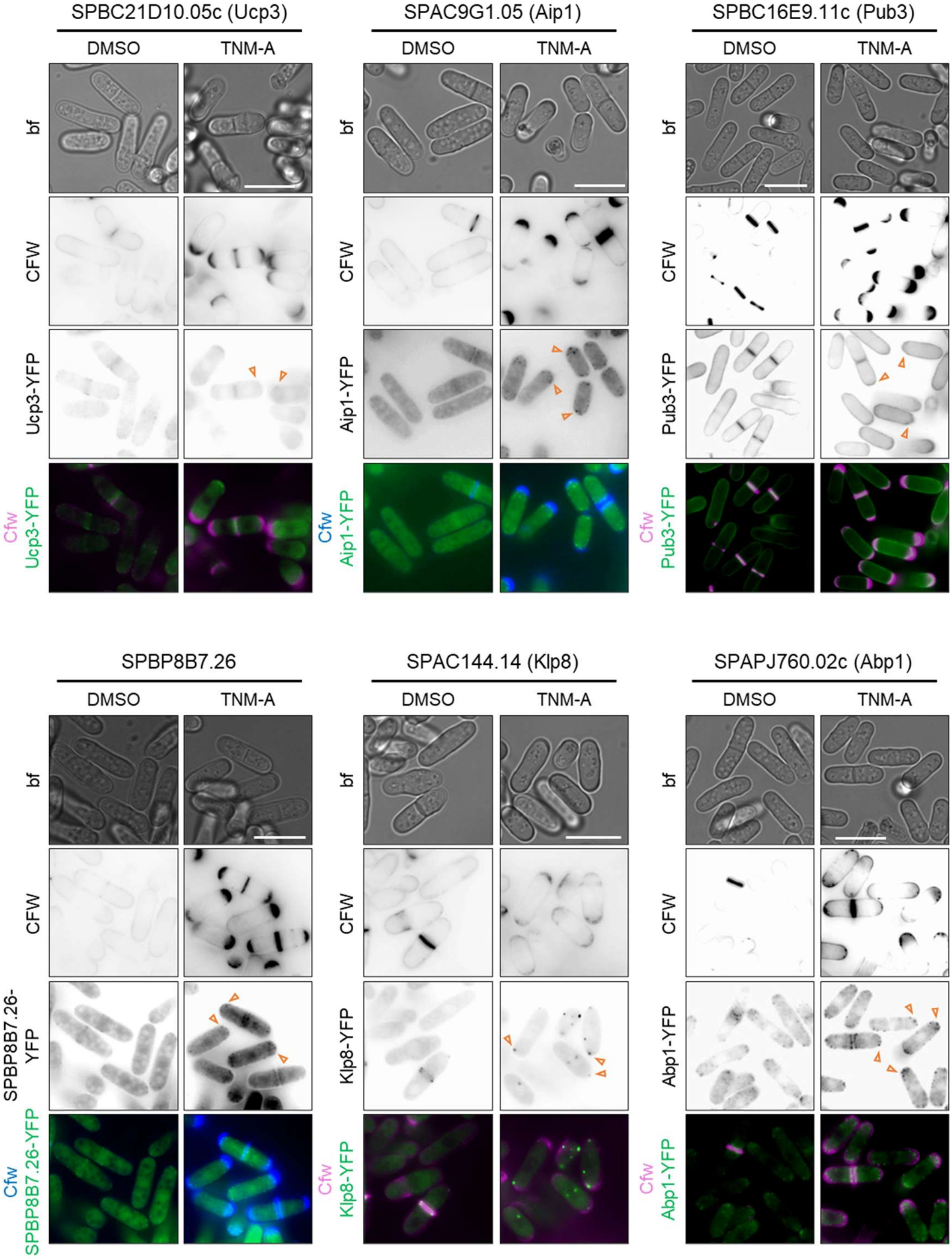

SPBC21D10.05c (Ucp3)
SPAC9G1.05 (Aip1)
SPBC16E9.11c (Pub3)
DMSO
TNM-A
bf
CFW
Ucp3-YFP
Cfw
Ucp3-YFP
Aip1-YFP
Cfw
Aip1-YFP
Pub3-YFP
Cfw
Pub3-YFP
SPBP8B7.26
SPAC144.14 (Klp8)
SPAPJ760.02c (Abp1)
SPBP8B7.26-YFP
Cfw
SPBP8B7.26-YFP
Klp8-YFP
Cfw
Klp8-YFP
Abp1-YFP
Cfw
Abp1-YFP

A-5

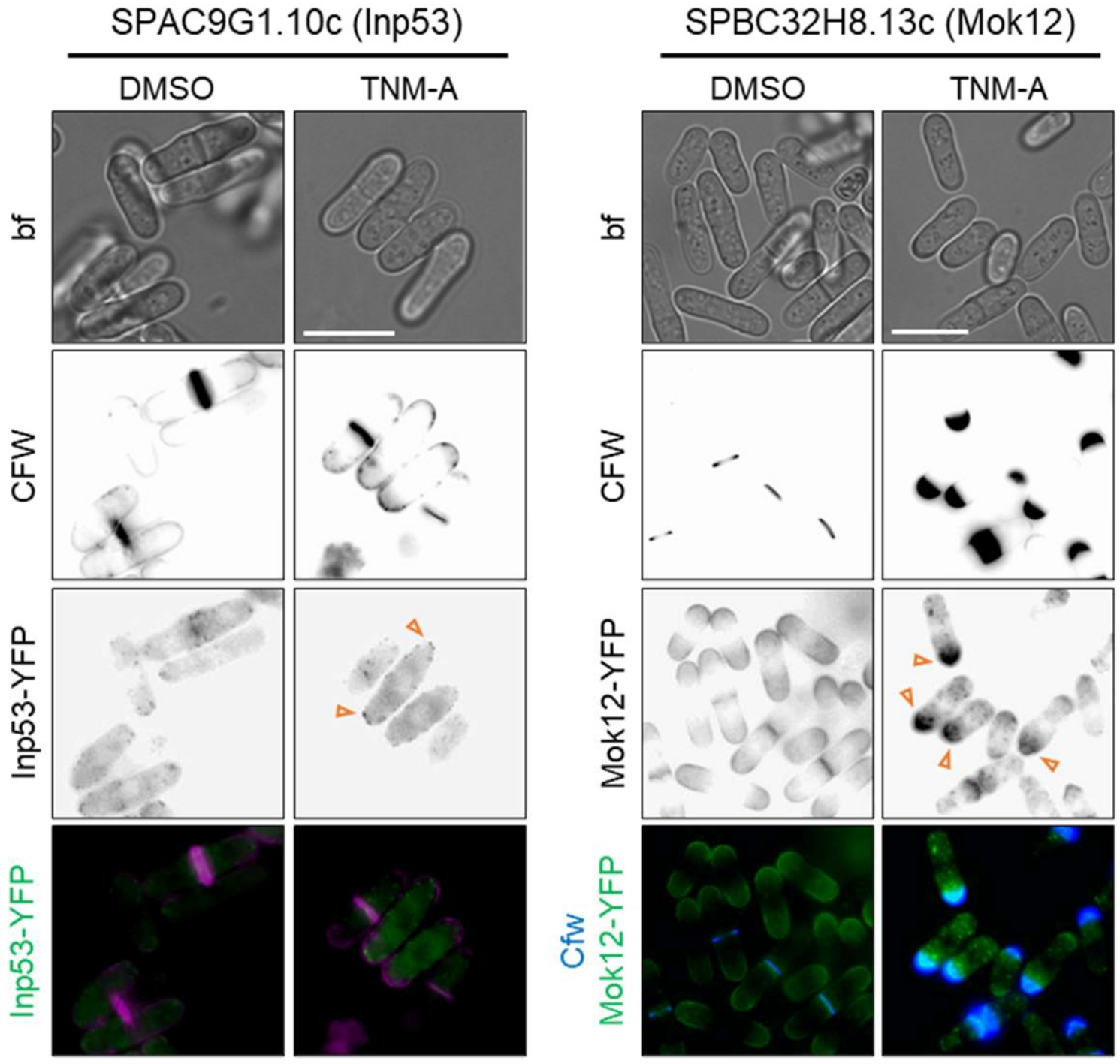
SPAC9G1.10c (Inp53)
DMSO
TNM-A
bf
CFW
Inp53-YFP
Cfw
Inp53-YFP
SPBC32H8.13c (Mok12)
DMSO
TNM-A
bf
CFW
Mok12-YFP
Cfw
Mok12-YFP

B

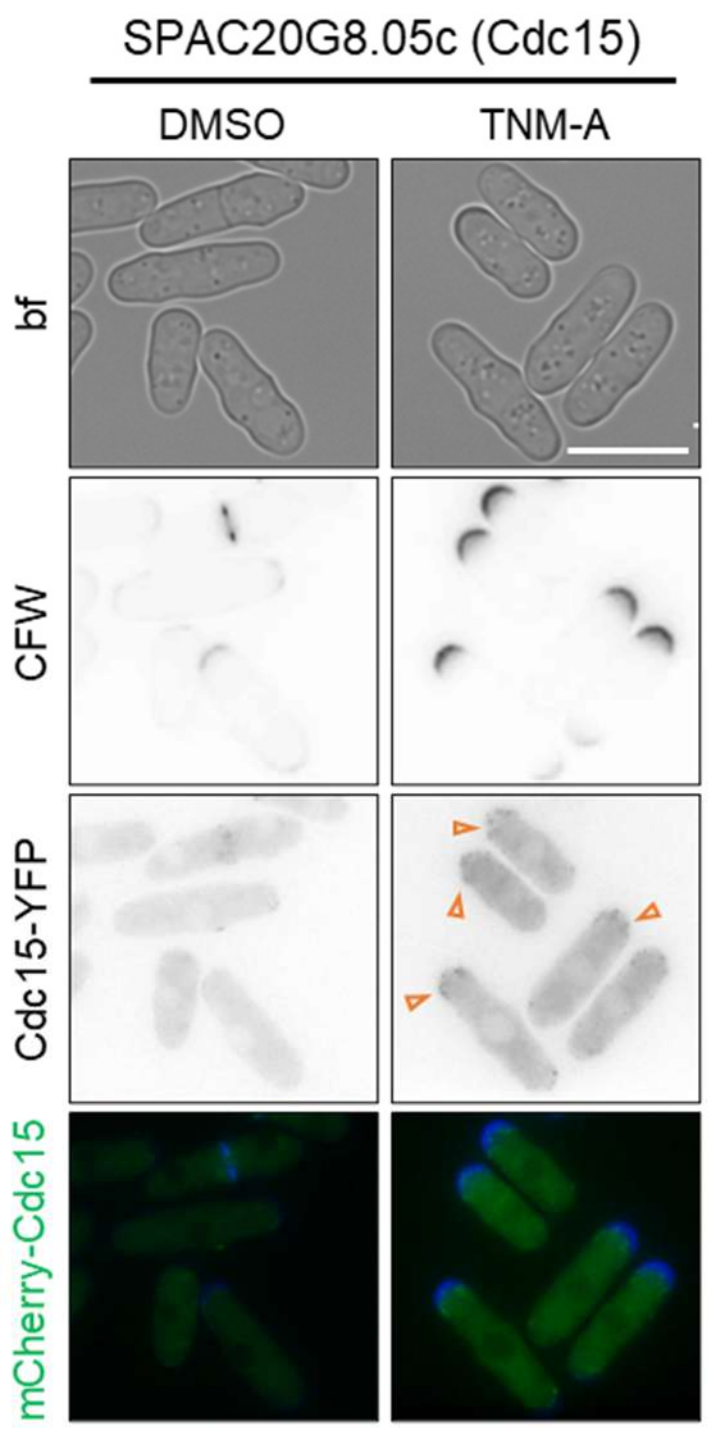
SPAC20G8.05c (Cdc15)
DMSO
TNM-A
bf
CFW
Cdc15-YFP
Cfw
mCherry-Cdc15

C

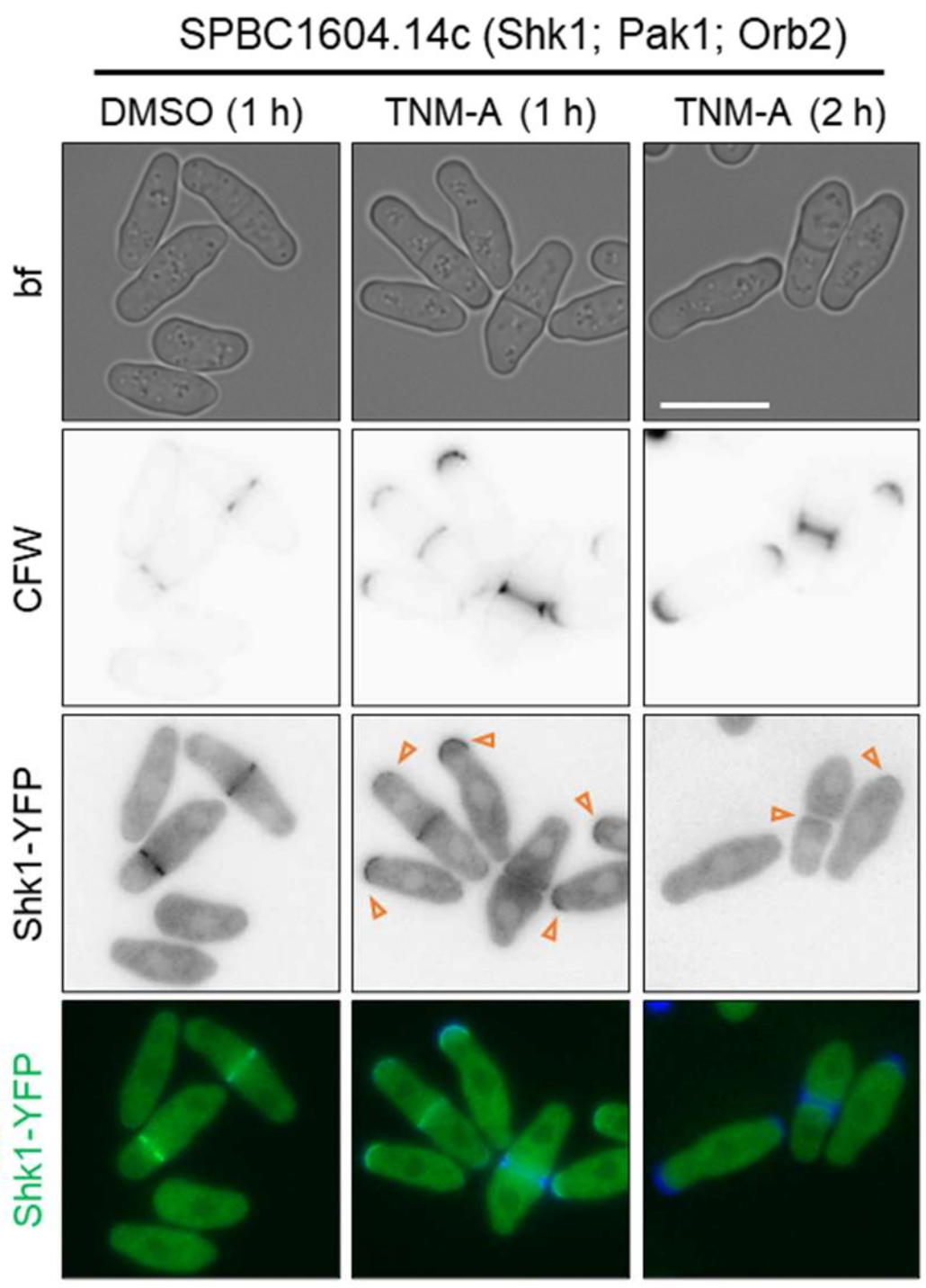
SPBC1604.14c (Shk1; Pak1; Orb2)
DMSO (1 h)
TNM-A (1 h)
TNM-A (2 h)
bf
CFW
Shk1-YFP
Cfw
Shk1-YFP

Data S1. Results of the localizome screen, related to Figure 4.

Subcellular localization of the proteins that are localized at/around thick cell wall are shown (orange arrowhead). Cells expressing YFP-tagged proteins under the control of the *nmt1* promoter or cells expressing mCherry-Cdc15 from the endogenous promoter were treated with DMSO or TNM-A (2 μg/ml) for 2 h (A1-A5, B). Cells expressing YFP-tagged Shk1 were challenged to DMSO or TNM-A (2 μg/ml) for 1 or 2 h. Cells were imaged for bright field (bf) and calcofluor white (CFW) and YFP fluorescence microscopy. Scale bars, 10 μm.
